# Effects of a novel loss-of-function waxy endosperm allele on sorghum seed development and grain quality

**DOI:** 10.1093/g3journal/jkaf177

**Published:** 2025-08-06

**Authors:** Pallavi Dhiman, Shejal Soumen, Deepti Nigam, Scott R Bean, Xiaorong Wu, Gunvant B Patil, Zhanguo Xin, Yinping Jiao

**Affiliations:** Department of Plant and Soil Science, Institute of Genomics for Crop Abiotic Stress Tolerance (IGCAST), Texas Tech University, Lubbock, TX 79409 United States; Department of Plant and Soil Science, Institute of Genomics for Crop Abiotic Stress Tolerance (IGCAST), Texas Tech University, Lubbock, TX 79409 United States; Department of Plant and Soil Science, Institute of Genomics for Crop Abiotic Stress Tolerance (IGCAST), Texas Tech University, Lubbock, TX 79409 United States; Grain Quality and Structure Research Unit, Center for Grain and Animal Health Research, USDA-ARS, 1515 College Ave, Manhattan, KS 66502, United States; Grain Quality and Structure Research Unit, Center for Grain and Animal Health Research, USDA-ARS, 1515 College Ave, Manhattan, KS 66502, United States; Department of Plant and Soil Science, Institute of Genomics for Crop Abiotic Stress Tolerance (IGCAST), Texas Tech University, Lubbock, TX 79409 United States; Plant Stress and Germplasm Development Unit, Crop Systems Research Laboratory, USDA-ARS, 3810, 4th Street, Lubbock, TX 79424 United States; Department of Plant and Soil Science, Institute of Genomics for Crop Abiotic Stress Tolerance (IGCAST), Texas Tech University, Lubbock, TX 79409 United States

**Keywords:** waxy sorghum, grain quality, seed development, starch biosynthesis, transcriptome, metabolome

## Abstract

Waxy sorghum seeds, defined by reduced amylose content in starch, offer the potential for improving grain quality in food and industrial applications. While waxy endosperms arising from a nonfunctional *waxy* (*wx*) allele leading to the absence of granule-bound starch synthase enzyme have been identified in sorghum, their broader effects on seed development and grain quality remain inadequately understood. To address this gap, we identified a novel *wx* loss-of-function allele, “*wx^e^*” in the mutant population of the sorghum reference genome line BTx623. Beyond reduced amylose content, *wx^e^* exhibited increased kernel hardness, elevated protein content, reduced endosperm-to-germ ratio, and decreased kernel weight compared to the wild-type. Integrating transcriptomic, metabolomic, and seed chemistry analyses revealed coordinated regulatory changes during seed development due to disrupted amylose synthesis. This included altered starch granule structure, enhanced lipid profiles, and reduced carbohydrate content. Differentially expressed genes and transcription factors related to starch metabolism provided insights into the regulatory mechanisms. Furthermore, metabolic profiling showed significant changes in the accumulation of compounds influencing flavor and nutritional properties. This study enhances our understanding of the molecular coordination of sorghum seed development and provides new insights into regulating seed development.

## Introduction

In cereal crops, such as maize, rice, wheat, barley, and sorghum, starch is an important source of carbohydrates and is used in human food, livestock feeding, and ethanol production for brewing and industry. Waxy grains with glutinous texture have been known to have enhanced gelatinization properties, higher free amino nitrogen, higher starch and protein digestibility, low viscosity during liquefaction, lower retrogradation, and shorter fermentation times (Zhao et al. 2009; Mezgebe et al. 2018; Cesevičienė et al. 2022).

The waxy endosperm results from mutations in the starch synthesis pathway genes (Huang et al. 2021). Starch primarily comprises 2 types of polymers: linear amylose and branched amylopectin (Jane 2004; Tetlow and Bertoft 2020). The starch biosynthesis process in plastids is mainly carried out by 5 enzymes, ADP-glucose phosphorylase (AGPase), starch synthase (SS), starch branching enzymes (SBEs), starch debranching enzymes (DBE), and granule bound starch synthase (GBSS). AGPase converts glucose-1-phosphate to ADP-glucose, which is the key substrate for starch synthesis (Zeeman et al. 2010; Seung and Smith 2019). Amylopectin synthesis is carried out by SS, SBEs, and DBEs. Amylose synthesis is conferred by the *GBSSI* gene in Sorghum. The polyploid crops such as wheat have 2 isoforms of *GBSS* gene: *GBSSI*, present in endosperm, and *GBSSII* present in leaf and maternal tissue of developing seeds (Jiang et al. 2003; Lü et al. 2008; Chen et al. 2016a). In cereal crops, it is well-studied that the mutations in the *GBSSI* disrupt the synthesis of amylose resulting in the waxy endosperm (Huang et al. 2021). As such, the *GBSSI* gene has been referred to as “*waxy*” gene in many studies.

Molecular characterization of *waxy* alleles (*wx*) at the *waxy* locus in the grass species has been extensively carried out. In rice, a G to T mutation alters the 5′ splice site of the first intron of the *waxy* gene leading to nonfunctional GBSSI enzyme (Bligh et al. 1998; Olsen and Purugganan 2002). In wheat, deletions have been identified in the 3 *GBSSI* loci, named *Wx-A1*, *Wx-D1*, and *Wx-B1* (Nakamura et al. 1993). Wheat lines with 1 or 2 *GBSS* genes mutated are known as partial waxy mutants and where all 3 genes mutated are completely amylose free (Gaur et al. 2024). In barley, the waxy genotype contains a spontaneous mutation in its 5′ noncoding sequence of *HvGBSSI* gene. Additionally, the induced *waxy* mutant SH97 has a stop-gained substitution in exon 5 triggering nonsense-mediated mRNA decay (Domon et al. 2002; Hebelstrup et al. 2017). In the waxy barley variety “*CDC Alamo*,” the amino acid substitutions D219 V, M490 V, and I491 V cause improper targeting of *HvGBSS1a* into starch granules due to 90% reduction in catalytic activity (Hebelstrup et al. 2017).

Similarly, 4 alleles in the sorghum *GBSSI* gene *Sobic.010G022600* were already reported to exhibit a waxy phenotype. *Waxy* allele is designated as *wx^a^* with large insertion in the third exon and *wx^b^* contains a missense mutation in the *SbGBSS* gene causing waxy phenotype (Pedersen et al. 2005; Sattler et al. 2009). Later another study identified 2 new *waxy* allele mutations in Chinese waxy sorghum, designated as *wx^c^* and *wx^d^* (Lu et al. 2013). The *wx^c^* allele involves a G deletion at the 5′ splicing site of the ninth intron, while the *wx^d^* allele features a G to C mutation at the 3′ splicing site of the 10th intron. Several studies (Pedersen et al. 2005; Sattler et al. 2009) showed that in *wx^b^* GBSS activity was reduced, whereas no GBSS activity was present in *wx^a^.* The amylose content in BTx630 (*wx^a^*), Tx2907 (*wx^a^*), B.9307 (*wx^b^*), and BTxARG1 (*wx^b^*) was found to be 1.5%, 1.3%, 1.1%, and 1.8%, respectively (Pedersen et al. 2005). Similarly, the amylose content of *wx^c^* line identified from Taiwanese landrace was found to be 0.71% (Kawahigashi et al. 2013).

Waxy grain sorghum with low amylose is gaining growing interest as the sorghum industry seeks novel applications for its grain in both food and industrial sectors. Waxy sorghum lines, especially white, tan-plant variants, demonstrated superior malt quality with enhanced endosperm modification, and starch granule degradation (Mezgebe et al. 2018). However, studies have evaluated that sorghum hybrids with waxy endosperm generally yield 10 to 17% less than nonwaxy hybrids (Jones and Sieglinger 1952; Tovar et al. 1977; Rooney et al. 2005). On the other hand, some waxy lines have shown comparable performance, indicating that high-yielding waxy genotypes can be developed (Rooney et al. 2005). However, the exact cause of the yield reduction in waxy hybrids is unclear and could result from pleiotropy or linkage between the *wx* gene and other genes that affect grain yield. Another study (Yerka et al. 2016) evaluated the agronomic performance of near-isogenic Wheatland × Tx430 hybrids with waxy, heterowaxy, or wild-type (WT) endosperm. The *wx^b^* × *wx^a^* hybrid with the lowest amylose produced more yield than the WT × WT hybrid, and the *wx^b^* × *Wx* hybrid had the highest yield, surpassing the WT (*P* < 0.05). These results highlight the importance of incorporating diverse waxy alleles in sorghum breeding to develop high-yielding waxy hybrids and sustain heterosis.

Recently, it was demonstrated that starch and fatty acid synthesis precede protein synthesis in sorghum seeds, raising the question of how alterations in one pathway may impact the carbon allocation of others (Khan et al. 2024). While it is established that loss of function in *GBSSI* results in waxy endosperm, the effects of amylose reduction on grain quality and seed development in sorghum are not well understood at the molecular level.

To address this question, we isolated a new *waxy* loss-of-function sorghum mutant and compared the seed development and grain quality using transcriptome and metabolome approaches. This work aims to fill a critical knowledge gap in understanding the molecular and biochemical basis of the waxy trait. The insight gained will set up the foundation to improve the sorghum grain quality to meet market needs. Additionally, the identification of novel alleles or genetic variants linked to the *waxy* gene may support the development of enhanced sorghum varieties with more desirable functional and nutritional traits.

## Materials and methods

### Plant material and sample collection

The study used the *Sorghum bicolor* reference genome line BTx623, and its ethyl methane sulfonate (EMS) induced mutant line 15M2-1184 (Jiao et al. 2016, 2024; Xin et al. 2023, 2008). The WT BTx623 and *waxy* mutant lines were planted at the Quaker Research Farm in Lubbock, Texas (33°35′52.9″N 101°54′21.4″W, elevation 992 m). The field has a semi-arid climate, with a yearly average rainfall of about 469 mm, and is characterized by Amarillo soil type. The 2 lines were grown in adjacent rows to ensure identical growth conditions with irrigation maintained at 1 inch of water per week. The developing sorghum seeds were collected with 2 replicates at 6, 11, 16, and 21 d postanthesis (DPA). The panicles were covered with pollination bags to avoid cross-pollination. Following pollination, the bags were replaced with mesh bags to prevent mold caused by humidity and allow adequate light penetration. The middle portion of 3 panicles that flowered on the same day were harvested for each replicate. The panicles were snap-frozen and stored at −80 °C for further analysis. Later, the seeds from the central part of the panicles were dissected on dry ice using scalpels and tweezers to isolate the endosperms.

### Genotyping of the waxy mutant

Leaf tissues were collected to isolate genomic DNA from immature plants nearly after 1 mo of germination. DNA was prepared by a CTAB-based method (Xin and Chen 2012). Allele-specific PCR was used to validate the existence of genetic mutations and identify individuals with homozygous mutations. The PCR was performed using the Allele Competitive Extension (PACE) master mix according to the manufacturer's protocol (3CR Bioscience, Harlow, United Kingdom) using a CFX96 Touch Deep Well Real-Time PCR Detection System (BIO-RAD, USA). PACE-PCR operates based on primers with distinct tail sequences designed to bind to known single nucleotide polymorphisms (SNPs), targeting specific alleles. The reaction mixture comprised 0.15 μL of the assay mix (containing 12 μM of each allele-specific forward primer and 30 μM of a single common reverse primer), 1 μL of gDNA (100–200 ng), 3.85 μL of ultra-pure distilled water, and 5 μL of PACE 2.0 Genotyping Master Mix (3CR Bioscience), bringing the total volume to 10 μL. The DNA was initially denatured at 94 °C, followed by a touchdown annealing step (65–57 °C) over 10 cycles for primer binding and initial extension. This was followed by 30 additional cycles for extended amplification to produce more tail-specific sequences. Fluorescence was then visualized and digitally recorded for automated analysis using Bio-Rad CFX Maestro software. In this study, tail sequences were incorporated into the forward primers for allele-specific genotyping. Specifically, FAM (5′-GAAGGTGACCAAGTTCATGCT-3′) was added to the forward primer for allele 1 (WT), and HEX (5′-GAAGGTCGGAGTCAACGGATT-3′) was added to the forward primer for allele 2 (mutant). The forward primer 1, forward primer 2, and common reverse primer are GGCTGAGCAGCACGTTCTC**C,** AGGCTGAGCAGCACGTTCTC**T,** GCAAAATAATTCAGGGCCCTGCCAA, respectively. The protein structure due to SNP change in *waxy* mutant and WT was predicted using Phyre2 (Kelley et al. 2015) and ChimeraX-v1.7.1 was used for visualization (Pettersen et al. 2021).

### Kernel characterization

The Single kernel characterization system (SKCS) was used to measure seed physical attributes viz. average hardness index, average kernel weight, and average kernel diameter (Bean et al. 2006). For kernel hardness index 3 replicates of 100 kernels each were analyzed. Seeds were dissected into 2 halves longitudinally and observed under a digital microscope to image the internal kernel structure. Vitreosity was measured using the image of 10 kernels from each line (Arthur et al. 2020). Density was measured using an Anton Paar Ultrapeer 5000 gas pycnometer.

Starch granule structure was visualized by scanning electron microscope (SEM) Hitachi S3400 (Phenom Pharos, ThermoFisher Scientific, Waltham, MA, USA and S4300, Hitachi, Ltd., Tokyo, Japan) using cryo stage setting for fresh developing seeds samples. Zeiss crossbeam 540 FIB-SEM (Carl Zeiss Microscopy, Germany) was used to observe the structure of the endosperm of the mature seeds. The specimens were affixed onto double-sided carbon tape, and Sputter-coated with iridium using the Safematic CCU-010 compact coating unit. The images were taken at 11.5 mm working distance with a 10 kV beam acceleration at different magnifications. The size of starch granules was measured using ImageJ software (Rueden et al. 2016). Developing seeds were freeze-fried, cut into 2 halves and fixed in 0.1 M phosphate buffer (pH 7.4) containing 2.5% glutaraldehyde (v/v) as described by Yan et al. (2024). The samples underwent dehydration by series of ethanol viz 30, 50, 70, 90, 100% (v/v). Thin sections of samples fixed in paraffin wax using a microtome were cut. Light microscopy images of developing seeds stained with Lugol solution and Coomassie blue were captured using Olympus BX53 microscope (Yan et al. 2024).

### Protein and starch analysis

The protein concentrations of the samples were assessed using the laboratory nitrogen combustion method, using a LECO FP828 with a nitrogen to protein conversion factor of 6.25 (AACCI Method 46–30.01) recognized as the standard reference technique, and adjusted to “dry basis” protein levels based on the grains’ moisture content (Paudel et al. 2018). The moisture of ground samples was determined using a Perten DA-7250 near-infrared spectrometer via an in-house moisture calibration curve (Paudel et al. 2018; Cuevas et al. 2023). The samples from 6, 11, 16, and 21 DPA and mature seeds were used for starch and amylose assay. Whereas 11, 16, 21 DPA and mature seed samples were used for kafirin analysis since protein accumulation is not apparent at 6 DPA. Seeds were lyophilized and ground into a fine powder using a mortar and pestle. Kafirin content was measured using reverse-phase high-performance liquid chromatography (RP-HPLC) (Bean et al. 2011). Total starch content and apparent amylose contents in the ground meal (Udy mill with 0.5 mm screen) samples were measured following the procedures described by (Peiris et al. 2021). Total starch was measured calorimetrically using a commercially available Megazyme K-TSTA kit (Bray, Ireland) following the example procedure (b, RTS-NaOH Procedure) (McCleary et al. 2019). Briefly, 100 mg grain meal in 16 × 120 mm glass tubes was wetted with 0.2 mL of 80% ethanol and dissolved in 2 mL NaOH (1.7 M) for 15 min. After mixing the dissolved samples with 8 mL, pH 3.8 sodium acetate buffer (pH adjusted to 5.0), and the samples were hydrolyzed at 50 °C for 30 min with α-amylase and amyloglucosidase (0.1 mL each). After centrifuging at 1300 rpm for 5 min, 0.1 mL of the hydrolysate was mixed with 3.0 mL GOPOD reagent and incubated at 50 °C for 20 min. The absorbance at 510 nm against the reagent blank was taken and used to calculate starch content. The apparent amylose content in the sorghum samples was quantified by measuring the absorbance of the polyiodide–amylose complex at 620 nm (Chrastil 1987; Peiris et al. 2021). Starch including amylose was dissolved in 90% DMSO:0.6 M urea solution at 100 °C on a heat block for 30 min. The dissolved amylose was complexed with iodine (I_2_–KI solution) in an acidic condition (0.5% trichloroacetic acid) for 30 min. Finally, the A_620_ was read against a reagent blank and used to calculate the apparent amylose from a standard curve built from reference amylose (potato, Megazyme # P-AMYL, Bray, Ireland) and amylopectin (maize, Sigma #10120, St. Louis, MI, USA).

### Metabolomic analysis

Untargeted metabolomic profiling was employed to analyze sorghum seeds using the Ultra-performance liquid chromatography–mass spectrometry (UPLC-MS) platform. For sample preparation, 35 mg powder of each sample was weighed into 1.5 mL Eppendorf tubes and immersed in a precooled extraction solution (75% methanol), supplemented with 20 μL of Internal Standard. Homogenization was conducted using a weaving grinder, followed by water bath ultrasonication at 4 °C for 20 min. After standing still at −20 °C for 1 h, the extracts underwent centrifugation at 14,000 rpm at 4 °C for 15 min. The resulting 20 μL of the supernatant solution from each sample was composited into the mixed quality control (QC) sample to assess the repeatability and stability of LC/MS analysis (Abbiss et al. 2020)

A Waters 2777c UPLC system paired with a Q Exactive HF high-resolution mass spectrometer (Thermo Fisher Scientific, USA) was employed for the separation and detection of metabolites. Raw MS data files (.raw) were converted into the.mzXML format using the Proteowizard MSConvert ver.3.0 tool with an inclusive MS level filter (Kessner et al. 2008; Chambers et al. 2012). Metabolite quantification was achieved by analyzing signal intensities through the XCMS-CAMERA data processing pipeline (Tautenhahn et al. 2008; Kuhl et al. 2012). To capture potentially rare metabolites within this diverse sample set, a minimum sample threshold of 3 was applied during the grouping phase of XCMS. Missing values were handled using imputation and interpolation methods to ensure data completeness, specifically through median imputation to reduce their impact on data structure while preserving usable data. For initial assessments of chemical diversity, LC-MS data from WT and *waxy* mutant samples were processed collectively. Mass features identified by XCMS-CAMERA were filtered based on retention times (60 to 630 s) and precise masses (m/z < 0.5 at the first decimal point), with naturally occurring isotopes excluded. Despite a high rate of false annotations, MS adducts were retained to minimize the risk of misclassifying true metabolites. The quantification of mass features was adjusted for tissue fresh weight and normalized by the total ion concentration of each sample to account for technical variability. Later each mass feature was log-transformed to facilitate multivariate analyses. The dataset was further refined using interquartile range filtering and Pareto scaling before analysis. To avoid undefined results in fold change and log2 fold change calculations, zero peak values in either sample mean were replaced with 1 (ie *Peak Value* + 1). This adjustment prevents division by zero and undefined logarithmic values.

Additional statistical analyses and data visualizations were conducted using the ggplot2 package in R. The enrichment and pathway analysis were performed using MetaboAnalystR 4.0 (Pang et al. 2024) and visualized using the ggplot2 package.

### RNA isolation and transcriptome analysis

Total RNA was isolated using RiboPure RNA purification kit (Thermo AM1924) from the endosperm following the manufacturer's instructions. The quality and quantity of RNA was measured using a Multiskan SkyHigh Microplate Spectrophotometer (Thermo Scientific, A5110C). cDNA library construction and sequencing were performed following the DNBSEQ eukaryotic transcriptome protocols. In brief, after purification, the fragments underwent end repair and single nucleotide A addition, followed by adapter connection. PCR amplification was then performed using selected fragments as templates. Quality control involved quantification and qualification using an Agilent 2100 Bioanalyzer and ABI StepOnePlus Real-Time PCR System. RIN ≥ 6.5 28S/18S ≥ 1.0 was used as quality cutoff score. Each cDNA library was sequenced on an Illumina HiSeqTM 2000 system with paired-end protocols. To ensure data quality, both raw and cleaned data underwent QC assessment using FastQC (Andrews 2010). High-quality reads were aligned to the sorghum version 3 reference genome using STAR (Dobin et al. 2013). Variants from the RNA-seq data were called using BCFtools mpileup (Danecek et al. 2021) using reads with mapping and sequencing quality scores above 20. Variants shared between the WT and *waxy* samples were excluded to account for differences arising from the various sources of BTx623 seeds. The targeted mutation in the *GBSS1* gene was confirmed in all *waxy* samples. Remaining GC-to-AT mutations were considered background mutations and annotated using SnpEff. The fragments per kilobase of transcript per million mapped reads (FPKM) values representing gene expression levels were calculated using Cufflinks (Trapnell et al. 2012). Pearson correlation coefficients between biological replicates were calculated based on gene FPKM values, and replicates with a correlation exceeding 0.9 were selected for further analysis.

### Differential expression analysis

The differential expression was calculated by Cuffdiff using default parameters (Trapnell et al. 2012). Genes were considered expressed if they showed > 1 FPKM at least at one time point. The genes with FPKM > 1 in either WT or *waxy* mutant at the comparative time point were considered. Differentially expressed genes were considered significant if they met 2 primary thresholds: a false discovery rate (FDR)-adjusted *P*-value (*q* < 0.05) and an absolute log₂ fold change ≥ 0.58, corresponding to a 1.5-fold change in expression. All comparisons were conducted by calculating the fold change as the ratio of *waxy* mutant to WT. In every comparative analysis “Up” indicates genes that are upregulated in the *waxy* mutant showing positive log₂ fold change, while “Down” denotes genes that are downregulated in the *waxy* mutant those with negative log₂ fold change values.

### Gene ontology enrichment analysis

The gene ontology (GO) term enrichment analysis was performed using Fisher's exact test and adjusting the FDR cutoff < 0.05. GO terms were extracted from the NCBI annotation of *Sorghum bicolor*, retrieved with record AH114726 with the R package AnnotationHub (Morgan et al. 2019). GO analysis of biological function pathways was performed using 9064 Entrez IDs (Maglott et al. 2005).

### Clustering analysis

The gene expression clustering using the average FPKM values was performed with Mfuzz R package (Kumar and Futschik 2007). The fuzzy c-means algorithm with a soft partitioning approach was applied. This clustering allowed genes with similar expression patterns to group together. Functional enrichment analysis for each cluster was performed using ShinyGO (Ge et al. 2020), considering pathways with FDR < 0.05 as significant.

## Results

### A new sorghum waxy endosperm allele

**Fig. 1.**
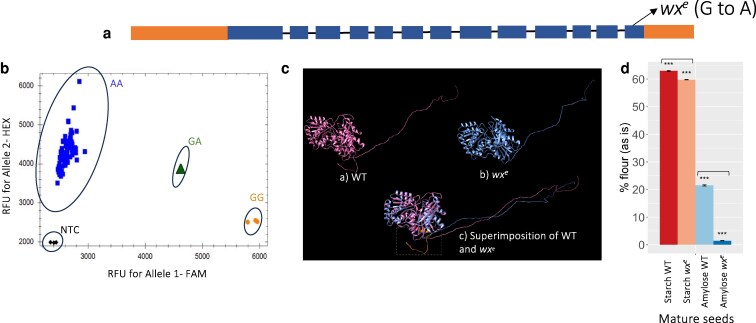
Identification of a new waxy sorghum allele. a) Gene structure of *GBSS1* (*Sobic. 010G022600*) highlighting the newly identified mutation. Exons and untranslated regions (UTRs) are represented by blue and orange boxes, respectively, with introns shown as connecting lines; b) Genotyping results detecting homozygous G to A mutation. The *x* axis represents relative fluorescence units (RFU) for FAM and *y* axis represents RFU for HEX; genotypic classification based on allele types: homozygous mutant lines (AA alleles) are represented by blue squares, heterozygous individuals (GA alleles) by green triangles, and WT (GG alleles) by orange circles, NTC is the negative control (water, no template); c) Predicted protein structures of WT and *wx^e^* mutant: a) WT structure shown in pink; b) *wx^e^* structure shown in blue; c) superimposition of WT and *wx^e^* structure. The region marked in orange (within the dotted box) indicates a key difference in protein conformation. d) Amylose and starch content change in mature seeds (***P < 0.001).

To understand the impact of reduction of the amylose synthesis on the sorghum seed development and other grain quality features, we obtained a new loss-of-function allele in the *GBSSI* gene from a sequence-indexed sorghum EMS mutant population line 15M2–1184 (Jiao et al. 2016, 2024). The mutation (G→A) in the *GBSS1* gene (*Sobic.010G022600*) is in the last exon of chromosome 10 at position 1864925 based on the version 3 sorghum BTx623 reference genome, leading to the change of amino acid tryptophan (W) to a stop codon (Fig. 1a). The homozygous mutants were confirmed by allelic specific primers using PCR. Allele-specific genotyping successfully differentiated the homozygous lines (blue), heterozygotes (green), and WT (orange), each forming distinct clusters, confirming clear allele discrimination (Fig. 1b). Only the seeds from homozygous mutant (AA) plants were used for further analysis. The protein structure indicated the absence of coil portion at the C-terminal in the mutant, altering the overall stability and function of the protein (Fig. 1c). To confirm the impact of the new allele on starch composition in the sorghum endosperm, we compared the content of amylose in the mature seeds of the mutant and WT. In WT seeds, the amylose content (% of flour as is), was 21.5%, whereas in the mutant, it was reduced to 1.47% (Fig. 1d). This result confirmed our identification of a new waxy allele in sorghum, named accordingly as “*wx^e^*”.

### Impact of reduced amylose on sorghum grain quality and seed development

**Fig. 2.**
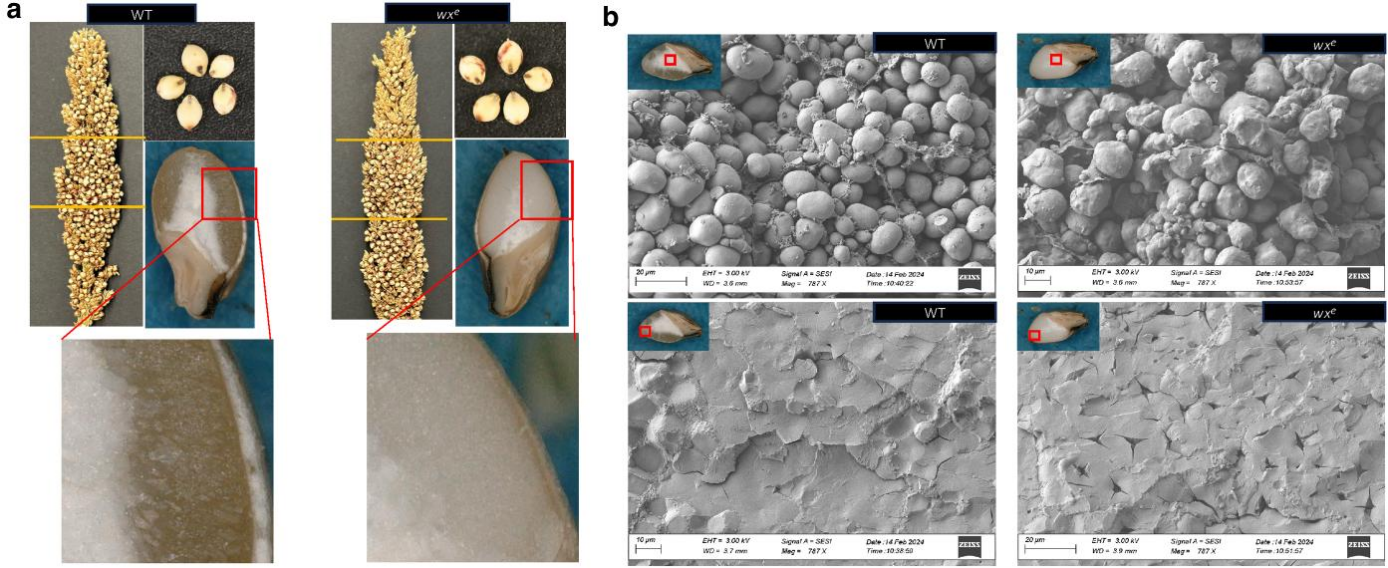
Seed physical characterization. a) Panicle and seed morphology and cross-section view of the endosperm of mature seeds; b) SEM imaging of floury and vitreous endosperm of the mature seeds.

The identical genetic background of our new waxy line and the reference genome line BTx623 enables a direct comparison, allowing us to assess the broader impact of knocking out the amylose synthesis on sorghum grain quality and seed development. Overall, we observed significant changes in the physical characteristics of mature *wx^e^* mutant seeds compared with the WT, including kernel weight, vitreousness and germ size, and hardness (Table 1). The mature seeds of *wx^e^* had ∼33% more vitreous endosperm than the WT. This vitreous characteristic in the *wx^e^* mutant is accompanied by a notably opaque or cloudy appearance (Fig. 2a). The mean kernel hardness of *wx^e^* mutant was increased by 5.29%, which is typically correlated with improved resistance to physical damage, prolonged storage stability, and suitability for long-term grain storage and export. Furthermore, the increased hardness in the *wx^e^* mutant is likely due to the expanded vitreous endosperm area and varying starch composition, while kernel density remains unchanged. The germ area showed a noticeable enlargement in the *wx^e^* mutant, measuring 2.77 mm² compared to 2.42 mm² in the WT. Conversely, the floury endosperm area reduced from 2.341 mm² in WT to 1.468 mm² in *wx^e^* mutant. Due to the increase in germ area, the overall endosperm to germ ratio was reduced in *wx^e^* seeds (1.78 vs 2.17). The overall total kernel area and diameter did not change significantly, indicating that waxy phenotype modifications within the seed did not impact the overall seed size (Table 1).

**Table 1.** Physical characterization of the mature seeds from the WT and *wx^e^*.

	WT	*wx^e^*
Average percentage vitreosity (%)	52.82	70.05^a^
Floury endosperm area (mm^2^)	2.341	1.468^a^
Average germ area (mm^2^)	2.42	2.77^a^
Average endosperm to germ ratio	2.17	1.78^a^
Average kernel area (mm^2^)	7.61	7.68^ns^
Average percentage germ (%)	31.84	36.03^a^
Mean Kernel hardness index^b^	71.54	75.32^a^
Mean kernel diameter (mm)	2.74	2.76^ns^
Mean kernel weight (mg)	32.45	30.27^a^
Density (g/cc)	1.35	1.35^ns^

ns, nonsignificant; g/cc, gram per cubic centimeter.

^a^Significant, *P* < 0.05.

^b^Crushing response profile of single kernel measured using SKCS.

The waxy seeds were characterized by altered starch and protein composition and resulted in reduced seed weight. WT exhibited a mean kernel weight of 32.45 mg, based on 3 replicates of 100 kernels each, while the *wx^e^* mutant showed a lower mean kernel weight of 30.27 mg. The total starch content was reduced by nearly 5% compared with the WT in the mature seeds (Supplementary Table 1). In addition, the total crude protein content in the mature seeds, calculated as protein (nitrogen × 6.25) on a dry weight basis, was higher in the *wx^e^* mutant (15.71%) compared to the WT (14.97%) (Supplementary Table 3). The increased total seed protein and reduced starch content in the *wx^e^* mutant indicate a change in resource allocation.

The starch granule structure of the *wx*^e^ mutant differed in size and shape compared with WT was observed (Fig. 2b). In the floury endosperm of *wx*^e^, the starch granules were nonuniform and rough/irregular in texture. In contrast, WT granules were round and smooth. As expected, in *wx^e^* vitreous endosperm, cracks appear due to loosely packed starch granules mainly composed of amylopectin (Fig. 2b). Amylopectin molecules tend to form a more open and porous structure compared to amylose (Cornejo-Ramírez et al. 2018). The average starch granule size in WT was 15.61 and 17.89 µm in the *wx*^e^ mutant in diameter attributed to the absence of amylose (Supplementary Fig. 1). This structural shift results in a more open and porous organization, which enhances accessibility to starch molecules, offering benefits during milling and cooking processes (Yang et al. 2024).

**Fig. 3.**
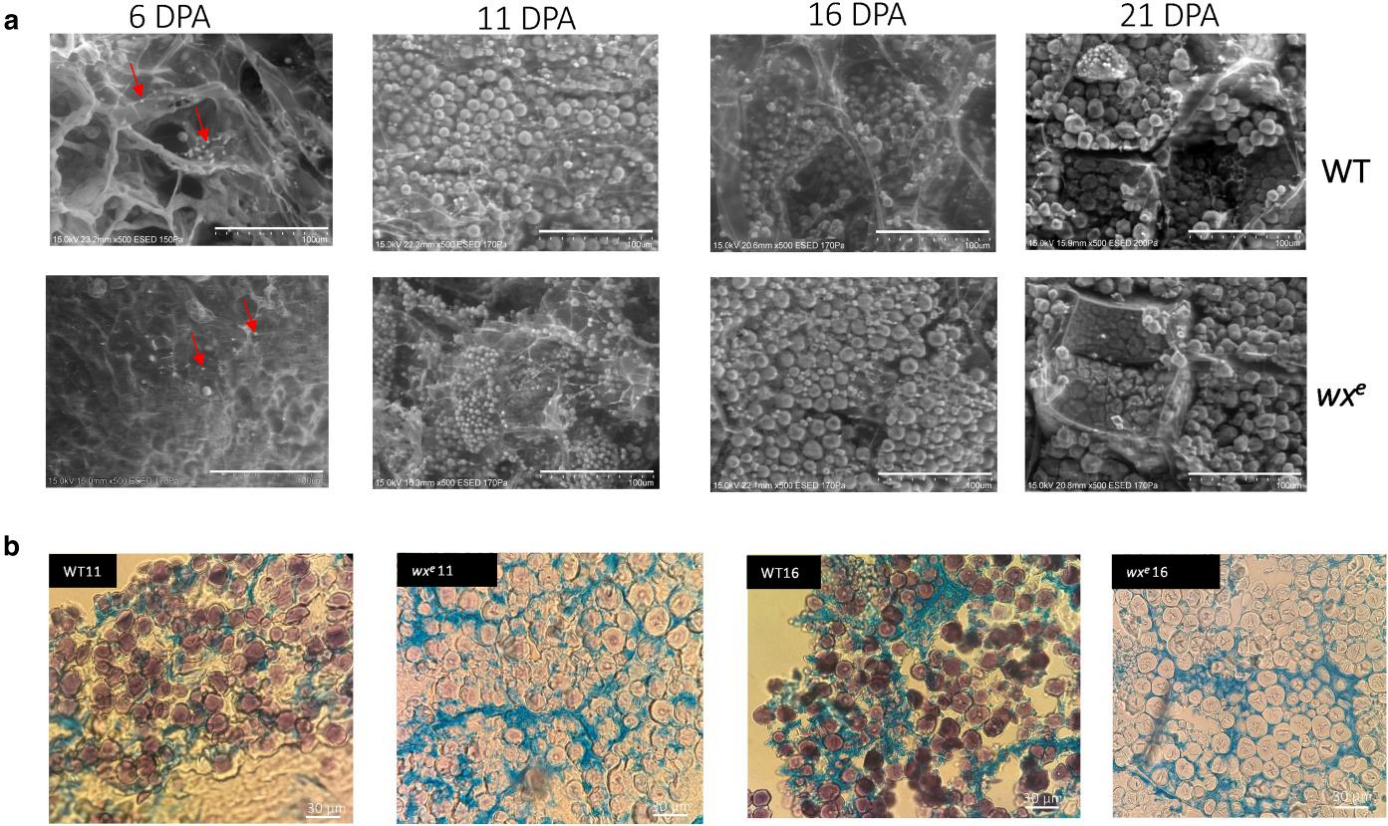
SEM imaging and starch staining of developing seeds. a) Cryo-stage SEM analysis of the floury endosperm at the 6, 11, 16, and 21 DPA in the WT and *wx^e^*; b) light microscopy imaging of semi-thin sections of developing seeds at 11 and 16 DPA stained with iodine and Coomassie blue observed at 100 × magnification.

To assess the impact of disrupting the amylose synthesis gene on sorghum seed development, we characterized the developing seeds of WT and *wx^e^* mutant at 6, 11, 16, and 21 DPA. These time points represent key developmental stages—milk, soft dough, and hard dough—during grain filling and correspond to major transcriptomic and metabolomic transitions (Khan et al. 2024). The SEM and seed cross-section staining analysis of developing seeds revealed a distinct pattern in starch and protein cross-linking, where waxy starch granules appeared to be loosely packed within the protein matrix (Fig. 3a). The starch granules of WT were stained uniformly dark with Lugol solution while *wx^e^* mutant starch grains were faintly stained (Fig. 3b). The uniform dark staining in WT starch granules indicates enriched amylose content, characteristic of normal starch structure. In contrast, the faint staining in *wx^e^* mutant reflects a significant reduction in amylose accumulation, consistent with quantitative data (1.47% vs 21.5% at maturity). Furthermore, during seed development, the amylose content in WT seeds increased from 0.07% at 6 DPA to 21.5% at maturity, whereas in the *wx^e^* mutant, it increased from 0.07% to a maximum of 1.95%. Moreover, the total starch content showed a consistent increase (% of flour as is), but at each time point, the *wx^e^* showed significantly less total starch content (Supplementary Table 1). The kafirin content which is the major storage protein in endosperm was low in *wx*^e^ only at 11 DPA (*P* < 0.05) but later did not show any difference (*P* > 0.05) (Supplementary Table 2). These results aligned with the changed starch and protein content in mature seeds.

### Lipid enrichment and the changed nutritional quality in *wx^e^* mutant

**Fig. 4.**
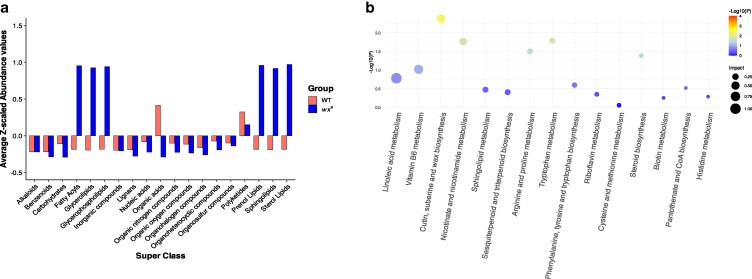
Comparison of metabolome profiling between the WT and *wx^e^*. a) Bar plot showing the average Z-scaled abundance of metabolites grouped by super-classes in WT and *wx^e^*. Values represent standardized compound levels, enabling comparison across classes and genotypes. Positive values indicate above-average abundance; negative values indicate below-average levels; b) top 15 upregulated metabolomic pathways in *wx^e^* mutant. The size of the dots represents the pathway impact, and the color represents the −Log10(*P*).

To further understand the change in grain quality, we performed metabolomic profiling of the mature seeds of WT and *wx*^e^. A total of 5,523 metabolite peaks were identified, and 1,265 were assigned to metabolite subclasses (Supplementary Dataset 1) in the metabolome of these 2 lines. A total of 599 and 440 were upregulated and downregulated with |log_2_FC| ≥ 1.0, respectively in *wx^e^*. The overall Z-scaled profiling of metabolite super classes revealed differences in the accumulation of specific classes, such as fatty acyls, glycerolipids, organic acids, and carbohydrates, between WT and *wxᵉ* (Fig. 4a), a pattern further supported by the clear group separation observed in the PCA plot (Supplementary Fig. 2a). The *wx^e^* line exhibited an enriched lipid profile including prenol lipids, sphingolipids, sterol lipids, glycerolipids and fatty acyl. Organic acids and carbohydrate classes showed low abundances in *wx^e^* (Supplementary Fig. 2b). The carbon metabolism pathways were downregulated in *wx^e^*, such as starch and sucrose metabolism, glycolysis, carbon fixation, pentose phosphate pathway, etc. The level of ADP-glucose was lower in *wx^e^* suggesting a reduced demand for the precursor of starch that the WT needs to synthesize amylose and amylopectin (Supplementary Dataset 1). Additionally, several pathways related to amino acid synthesis, including arginine biosynthesis, alanine, aspartate and glutamate metabolism, phenylalanine, tyrosine metabolism, etc., were downregulated in *wx^e^* (Supplementary Fig. 2d).

In contrast, specific fatty acid-related pathways such as linoleic acid metabolism, cutin, suberine, and wax biosynthesis upregulated in the *wx^e^* (Fig. 4b; Supplementary Fig. 2c). Additionally, the metabolic analysis revealed 185 benzenoids (log_2_FC > 1.0) were upregulated in *wx^e^*, and interestingly among those benzaldehyde, 2-aminobenzoic acid, and vanillic acid to name a few are known to impact flavor in the seeds. Other flavor-related compounds such as xylose (sugar and derivatives), feruloylputrescine (amino acids and organic acids), indoline (alkaloids) etc. (Luo et al. 2024) were upregulated in *wx^e^* (Supplementary Dataset 1). Overall metabolomic profiling of mature seeds from WT and *wx^e^* revealed significant metabolic differences, with *wx^e^* displaying an increase in lipid subclasses, and secondary metabolites and a reduction in organic acids, amino acids, and carbohydrate subclasses. These findings highlight the change in starch synthesis during seed development could also lead to a broader shift in the metabolic landscape, influencing not only primary metabolic pathways but also the accumulation of secondary metabolites and compounds associated with seed flavor and nutritional quality.

### Altered expression of starch and protein synthesis pathways in *wx^e^* mutant

Transcriptome profiling of endosperm was conducted at 4-time points—6, 11, 16, and 21 DPA as mentioned above with 2 replicates to understand the impact of impaired GBSS activity on the whole seed development. The correlation of the replicates of our data reached 99%, indicating high-quality (Supplementary Table 4). The homozygous mutation site was also validated using RNA-seq data (Supplementary Table 5).

Further, the whole-genome sequencing (WGS) of a segregating M3 pool (*n* = 20 individuals) derived from this line revealed a total of 6,231 mutations (Jiao et al. 2024). Given the pool-based sequencing approach, the individual M3 plant used in this study is expected to carry substantially fewer unlinked mutations. Complementary RNA-seq analysis, mapped to the BTx623 reference genome, identified 759 mutations in the mutant line. Out of 759 we found 211 amino acid chan,ges and large-effect mutations (stop-gained, splicing site change, and start lost), with 29 heterozygous and 182 being homozygous (Supplementary Datasets 2). Notably, the 15M2-1184 mutant harbored no large-effect mutations in known starch biosynthesis genes other than *waxy* gene. One gene *Sobic.004G163700* (*SbSBEIIb*) related to starch biosynthesis showed a nonsynonymous homozygous G261E mutation and downregulation of expression at 6 DPA. However, there was no expression change at later time points meaning the mutation is not overall impacting the gene expression. There were only 4 background genes with large-effect mutations that were downregulated in *wx^e^* at all time points: *Sobic.001G175000* (jmjC domain containing protein), *Sobic.001G501800* (C2H2 zinc finger protein), *Sobic.008G013000* (expressed protein), and *Sobic.003G017500* (tetratricopetide-repeat thioredoxin-like 3) (Supplementary Dataset 3). To the best of our knowledge, these genes are not known to impact endosperm starch and protein biosynthesis. This analysis confirms that the observed waxy phenotype and associated transcriptome changes are due to a single *wx^e^* stop-gained allele. Moreover, no significant difference in plant growth or morphology was observed between the mutant and WT under field conditions, supporting the suitability of this line for functional characterization.

**Fig. 5.**
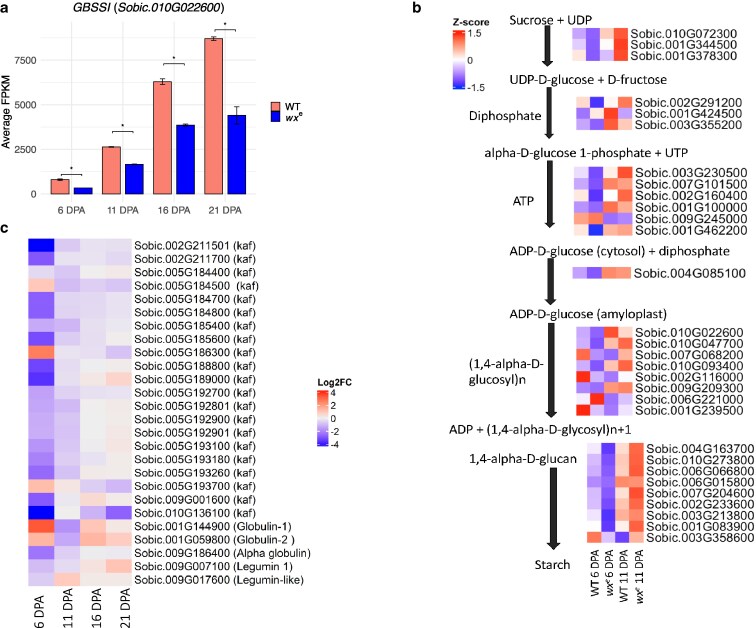
The altered expression of starch and protein pathway genes in the *wx^e^*. a) Expression levels of *GBSSI* (*Sobic.010G022600*) gene in WT and *wx^e^* at 6, 11, 16, and 21 DPA. Bars represent mean FPKM ± standard error (*n* = 2). The gene is differentially expressed at all time points between WT and *wx^e^* (|log₂FC| ≥ 0.58, *q* < 0.05) as determined by Cuffdiff analysis, indicated by “*.” b) Differential expression pattern of genes involved in the starch biosynthesis pathway, including *GBSSI* shown as a heatmap based on Z-score normalized expression values; c) differential expression pattern of genes associated with protein biosynthesis. Heatmap represents log2 fold change (log2FC) values, with color gradients indicating upregulation (red) and downregulation (blue). Gene names include kafirin (kaf), globulin, and legumin-like proteins.

Expression differences between the WT and *wx*^e^** mutant were more evident at early stages (6 and 11 DPA) than at the more mature stages (16 and 21 DPA) (Supplementary Table 6). This result aligns with the reported conclusion that grain filling primarily involves starch and protein biosynthesis, which is more active during the soft dough stage compared to the hard dough stage (Khan et al. 2024). In WT, 17,670 genes showed expression levels of FPKM > 1 at least one time-point, compared with 18,163 genes in *wx^e^* (Supplementary Dataset 4). As the *waxy* gene is part of the starch biosynthesis pathway, we first analyzed the expression of genes in starch biosynthesis. The *Sobic.010G022600* (*SbGBSSI*) gene expression was reduced in the *wx^e^* throughout the grain-filling stages due to the stop-gained mutation potentially causing nonsense-mediated mRNA decay (NMD), reducing the steady-state transcript levels (Zhang and Guo 2017) (Fig. 5a). Majority of starch biosynthesis genes, except for *Sobic.006G221000* (*Starch Synthase IIIb*, *SbSSIIIb*), which is involved in formation of amylopectin long chain, were significantly downregulated in *wx^e^* at 6 DPA. The shift toward amylopectin biosynthesis in *wx^e^* is indicated by the increased expression of most of the amylopectin biosynthesis genes such as *Sobic.001G239500* (*SbSSIIC*), *Sobic.007G068200* (*SbSSIIIa*), *Sobic.009G245000* (*SbAGPLS2*) after 6 DPA at different time points (Supplementary Dataset 5; Fig. 5b). Expression of genes like α-1,4-glucan phosphorylase L isozyme *SbPHOL* and *SbAGPS2* encoding glucose-1-phosphate adenylyltransferase appear to be significantly impacted by the *waxy* allele mutation at different developmental stages (Supplementary Dataset 5). Despite an increased expression of amylopectin synthesis-related genes to sustain overall starch content, the total starch content in *wx^e^* mutant is still lower than WT, as mentioned above. This may be due to the absence of efficient amylose synthesis, which reduces the total starch accumulation despite increased amylopectin production.

To investigate the increased crude protein in *wx^e^*, we first compared the expression of the major storage protein prolamin namely kafirin encoding genes in endosperm. At 6 DPA, kafirin genes, including 2 γ-kafirin, 7 α-kafirin, and 1 δ-kafirin genes, exhibit significant downregulation in *wx^e^* mutant (Fig. 5c; Supplementary Dataset 5). These results are consistent with the observed total kafirin content in seed which is significantly lower in *wx^e^* mutant compared to WT in the 11-day sample. In contrast, another sorghum storage protein globulin encoding genes (globulin-1[*Sobic.001G144900*] demonstrated upregulation at 6 and 16 DPA and globulin-2 [*Sobic.001G059800*]) at 6, 16, and 21 DPA. This upregulation may represent a compensatory response to maintain adequate storage protein levels despite the suppression of kafirins. These changes modified the interactions between starch granules and the surrounding protein matrix, affecting the structural properties of the endosperm and leading to the cloudy appearance of the vitreous endosperm in waxy seeds.

### Transcriptional regulation of sugars and flavor-related genes in *wx^e^* mutant

The precursor of starch synthesis in the endosperm of cereal grain is soluble sugar. To further uncover the genetic basis of the changed metabolome profile of *wx^e^* mutant endosperm, we evaluated the expression of genes involved in forming starch precursors. For instance, sucrose synthase 2, SS2 (*Sobic.001G344500*), sucrose synthase 3, SS3 (*Sobic.001G378300*), UTP glucose-1-phosphate uridylyltransferase (*Sobic.002G291200*), similar to sucrose transporter (*Sobic.001G488700*) were downregulated in *wx^e^* mutant at early grain development (6 DPA). SS2 showed upregulation at 11 and 21 DPA and SS3 at 11 DPA in *wx^e^* mutant. Most of the genes in the starch pathway were downregulated at 6 DPA in *wx^e^* and only a few including *SbAGPLS1* (*Sobic.003G230500)* at 11 DPA*, Sobic.002G160400* that converts α-D-glucose 1-phosphate to ADP-D-glucose at 16 and 21 DPA showed upregulation (Fig. 5b;  Supplementary Dataset 5). This indicates a low conversion of sugars, specifically sucrose, into starch. Other sugar transporters outside the starch biosynthesis pathway, including *SbSWEET1* (*Sobic.003G377700*) and *SbSWEET16* (*Sobic.001G377600*), exhibited significantly elevated expression in the *wx^e^* mutant relative to the WT at 6 DPA. Additionally, invertase 3 (*Sobic.004G004800*), which is only expressed at 6 DPA (FPKM >1), was upregulated in the *wx^e^* mutant. The *SbAGA1* (*Sobic.002G075800*) gene associated with raffinose metabolism and known for its role in flavor modulation, was upregulated in *wx^e^* mutant at 6 DPA while *SbAGA2* (*Sobic.002G075900*) did not show a significant difference at any time point (Supplementary Dataset 6). These observations suggest a coordinated regulation of carbohydrate metabolism pathways that influence grain quality traits in waxy seeds during early grain development, particularly in the absence of the *wx* gene.

### Upregulated fatty acid synthesis during the seed development in *wx^e^* mutant

**Fig. 6.**
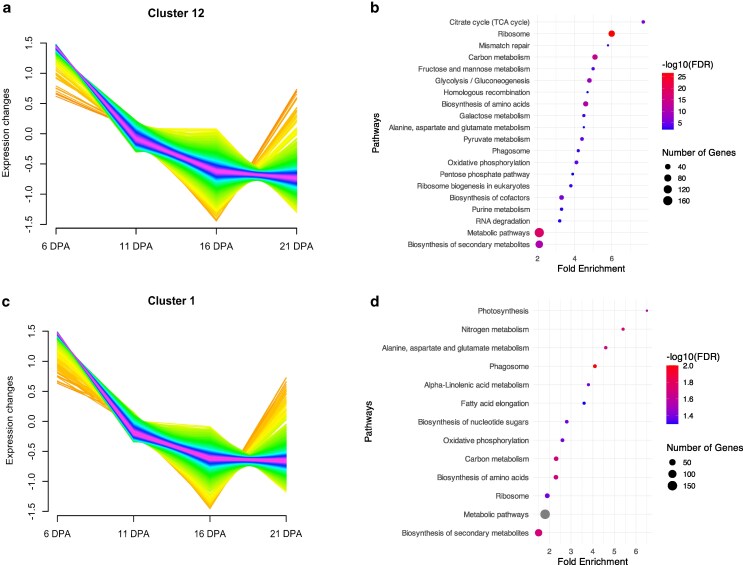
The coexpression cluster shows an expression pattern of a gradual decrease from 6 DPA to 21 DPA in the WT and *wx^e^*. a) The coexpression cluster with this pattern in the WT; b) pathways enriched in this cluster in WT; c) the coexpression cluster with this pattern in the *wx^e^*; d) the enriched pathways for the cluster in *wx^e^*. Colored trajectories represent expression changes, with each line corresponding to an individual gene's expression profile. The *x* axis represents time points 6, 11, 16, 21-DPA and the *y* axis represents log2-transformed normalized gene expression levels, ranging from −1.5 to 1.5. In enrichment plots, the *x* axis represents fold enrichment, while the *y* axis lists significantly enriched pathways. Dot size indicates the number of genes associated with each pathway, and color intensity represents the significance level (−log10[FDR]). The clustering was performed using the Mfuzz R package with a soft partitioning approach resulting in 12 clusters. The cluster enrichment analysis was performed using ShinyGO.

A coexpression network analysis revealed genes with similar expression patterns. Among the 12 coexpression modules, cluster 12 with 1,681 genes in WT and cluster 1 with 1,908 genes in the *wx^e^* mutant (FDR < 0.05) showed a similar expression pattern of a gradual decrease from 6 DPA to 21 DPA (Supplementary Dataset 7; Fig. 6a,c). Interestingly, the genes with the same expression pattern showed distinct functional categories in WT and *wx^e^* as obtained from GO analysis. For instance, the common pathways in WT and *wx^e^* mutant included carbon metabolism, biosynthesis of amino acids, nucleotide metabolism, metabolic pathways, ribosome biogenesis, and biosynthesis of secondary metabolites. Pathways such as nitrogen metabolism, alpha-linolenic acid metabolism, fatty acid elongation, and biosynthesis of nucleotide sugar were enriched exclusively in *wx^e^* mutant (Fig. 6b,d;  Supplementary Fig. 3a,c). Notably, no cluster in WT showed enrichment for fatty acid biosynthesis, suggesting that in the absence of amylose, carbon allocation in *wx^e^* may preferentially direct toward fatty acid or oil biosynthesis. Similarly, WT cluster 8 and *wx^e^* mutant cluster 11 have similar functional enrichment except for the absence of starch and sucrose metabolism in *wx^e^* (Supplementary Fig. 3b,d).

Pathways such as starch biosynthesis process, reproductive processing, and protein maturation among the top 20 pathways were downregulated in *wx^e^* mutant at 6 DPA indicating these pathways being less active in *wx^e^* mutant. Lipid localization was upregulated in *wx^e^* mutant at 16 DPA as compared to WT (Supplementary Dataset 8 and Supplementary Fig. 4a). A substantial upregulation was observed in fatty acid biosynthesis genes in the *wx^e^* mutant compared with the WT at 6 DPA (Supplementary Fig. 4b and Supplementary Dataset 9). The upregulation of genes involved in the early steps of fatty acid biosynthesis (eg 3-ketoacyl-CoA synthase 1, acyl-CoA oxidase 3) suggests an increased flux through the fatty acid biosynthesis pathway in the *wx^e^* mutant. Furthermore, the 3-ketoacyl-CoA synthase 1 (*Sobic.001G482300*) also showed upregulation at 16 and 21 DPA. The changes in fatty acid gene expression and composition might contribute to the observed differences in kernel phenotypic traits. Only malonyl CoA-acyl carrier protein transacylase (*Sobic.001G403400*) showed significant downregulation at 6 DPA. This indicates specific adjustments in the fatty acid metabolism, potentially affecting the balance of fatty acid elongation and modification processes. These findings suggest that the differences between *wx^e^* and WT extend beyond physical waxy traits, involving numerous genes associated with various primary and secondary metabolic pathways.

## Discussion

### Amylose reduction: starch metabolism, grain quality, and beyond

Seed development following pollination, which mainly includes starch and protein accumulation, is a highly coordinated and regulated process. In cereal grains, the interactions between amylose, lipids, and amylopectin contribute to distinct physicochemical properties critical for downstream applications of starch (Varghese et al. 2022; Sengupta et al. 2023). Elevated amylopectin levels (low amylose), a defining feature of waxy grains, facilitates hydration through hydrogen bonding, promoting gel formation, and retrogradation tendencies (Lan et al. 2008). Additionally, smaller starch granules, with increased surface area, pores, and channels, enhance water absorption. This increased hydration capacity leads to increased swelling, viscosity, and gelatinization potential of starch granules (Cornejo-Ramírez et al. 2018). However, in our *wx^e^* mutant, the starch granules were bigger in size than the WT, but they also had a porous structure. Waxy seeds are widely consumed due to their texture; however, the increased digestibility of amylopectin in the absence of amylose leads to a spike in blood sugar levels. Furthermore, the interaction among starch, lipids, and proteins leads to the development of highly structured ternary complexes in the seed endosperm (Naseer et al. 2021). In nature, waxy seeds, including inbred lines, exhibit differences not only in starch metabolism but also in other biological pathways, such as those involved in secondary metabolite production (Luo et al. 2024). Previous studies have shown the genetic basis as well as the expression pattern of starch biosynthesis including amylose and amylopectin in sorghum (Campbell et al. 2016). Our study offered a system investigation of the reduction of amylose biosynthesis pathway to sorghum seed development and grain quality using seed chemistry, metabolome, and transcriptome approaches. Multi omics analysis highlighted that the consequences of amylose biosynthesis disruption impact the overall seed development, not only starch. Notably, the loss-of-function of a single gene within the same genetic background resulted in variations that extended beyond the starch biosynthesis pathway, highlighting the broader biological impact of targeted genetic modifications. In gene expression analysis, the downregulation of most starch biosynthesis genes, including *SbGBSSI*, coupled with the upregulation of amylopectin synthesis-related genes, reflects a compensatory mechanism to sustain starch levels. Despite these efforts, the imbalance in amylose–amylopectin synthesis underscores the need for fine-tuned regulatory interventions to optimize starch yield while preserving quality traits. Further, new sources of waxy mutants containing mutations in the promoter region, or missense mutations in *GBSSI* could be explored. This could help to dissect the lines with less dramatic changes in the seed quality and obtain intermediate levels of amylose between WT and *wx*. Additionally, recent studies have identified protein targeting to starch 1 (PTST1) as a key regulator involved synthesis primarily through interaction with GBSS. Arabidopsis *ptsts1* mutants showed deficient amylose in leaves (Seung et al. 2017), However, its role in cereal endosperm remains unclear. For instance, rice knockout of *PTST1* gene resulted in amylose free starch in leaves with minimal impact on the endosperm starch composition (Wang et al. 2020). Contrastingly, in barley the knockout mutants produced seeds with no starch in the endosperm indicating its species-specific role (Zhong et al. 2019). Therefore, investigating the function and interaction of PTST1 in sorghum could provide valuable insights into its role, if any, in endosperm amylose regulation.

### Insight into transcriptional regulation of starch biosynthesis

Starch biosynthesis is a multifaceted process controlled by various genes, along with numerous transcription factors and mediators, which in turn influence grain weight, appearance, and overall seed composition and quality. Spatiotemporal-dependent regulators of starch biosynthesis in cereal crops particularly focusing on transcription factors offer new insights into its regulation. Studies on transcriptional regulation of starch biosynthesis have reported some transcription factors that bind the starch biosynthesis genes and regulate the gene expression (Sun et al. 2003; Zhang et al. 2014; Huang et al. 2016; Chen et al. 2016b; Xiao et al. 2022b). A total of 61 transcription factors (TFs) previously identified in sorghum (Xiao et al. 2022a) with FPKM > 1 at any time point were compared between the 2 samples. Among all TFs NAC, NF_YC, Dof, bZIP, bHLH showed very high expression levels in the endosperm (Supplementary Dataset 10 and Supplementary Fig. 5). We observed that 20 TFs related to starch biosynthesis were significantly downregulated in *wx^e^* at 6 DPA and 19 were downregulated at 11 DPA. Interestingly, NAC TFs *Sobic.003G105600*, *Sobic.009G086800*, *Sobic.002G192600*, and *Sobic.005G056300* have already been identified to bind to the Cis regulatory region of sorghum *waxy* gene (Xiao et al. 2022a) were significantly downregulated in *wx^e^* at 6 DPA. In rice, overexpression of *OsNAC129* resulted in the downregulation of the *OsGBSS1* gene, aligning with its role as a negative regulator of starch synthesis (Jin et al. 2022). Other TFs including bZIPs, ERFs, and MYBs have been characterized in cereals to be involved in the regulation of starch synthesis (Li et al. 2021). In our study, *Sobic.002G055000* and *Sobic.002G054800*, members of the bZIP transcription factor family, *Sobic.007G147300*, an ERF family member, and *Sobic.002G308400*, associated with the MYB transcription factor family, were downregulated in the *wx^e^* mutant at various time points. Notably, all these transcription factors showed consistent downregulation at 6 DPA. On the other hand, only a few TFs including *Sobic.009G170800* (ERF), *Sobic.003G267400* (MYB), *Sobic.005G019800* (ZF-HD) were upregulated in *wx^e^* at 6 DPA and *Sobic.007G083900* (B3) was upregulated at 16 and 21 DPA (Supplementary Fig. 5). There is limited understanding of starch regulation in sorghum at the transcriptional level. The differentially expressed TFs observed in the *wx^e^* compared to the WT may provide targets to further investigate the complex regulatory mechanisms controlling starch biosynthesis and offer potential targets for genetic manipulation. As also discussed above, the mutations in TFs binding sites of *GBSSI* genes could be explored to achieve precise regulation of *GBSSI* gene expression resulting in waxy traits.

### Future research perspective

The novel waxy endosperm mutant 15M2-1184, developed in the reference genome BTx623 genetic background, provides an excellent platform to study the broad functions of the GBSSI gene in seed development and grain quality. In contrast, previously reported sorghum waxy alleles were identified in distinct genetic backgrounds (McIntyre et al. 2008; Sattler et al. 2009; Lu et al. 2013), making direct comparisons with WT endosperm sorghum challenging. Looking ahead, additional backcrossing and molecular analysis will help refine the genetic background and reduce the number of unlinked mutations. Additionally, the precise genome editing eg clustered regularly interspaced short palindromic repeats and CRISPR-associated protein 9 (CRISPR/Cas9) can be leveraged to introduce favorable waxy alleles without extensive breeding cycles. Although *wx^e^* showed significantly less amylose consistent with waxy phenotype, the precise nature of GBSS protein accumulation and enzymatic activity remains to be elucidated. The impact of *GBSS1* mutation on protein accumulation or enzymatic activity will help to classify *wx^e^* as a complete or partial loss-of-function allele. Another promising avenue is the systematic investigation of allelic interactions among different waxy alleles. These studies will facilitate the comparative analysis of waxy alleles to assess their functional contribution, potential dosage-dependent effects, and possible pleotropic interactions. As mentioned earlier in the introduction section, studies have shown that the waxy hybrids showed changes in the yield as compared to nonwaxy lines (Rooney et al. 2005; Yerka et al. 2016). Some waxy inbred lines with yields comparable to the best nonwaxy lines have been identified suggesting that the negative yield association is likely due to genetic linkage (Rooney et al. 2005). Breaking the linkage through recombination and selection could lead to the development of high-yielding waxy hybrids. This new source of waxy sorghum (*wx^e^*) provides an opportunity to generate new waxy hybrids while maintaining hybrid vigor, rather than using the same allele in both parents, which can reduce heterosis. Investigating allelic interactions, combined with strategic recombination and selection, may facilitate the identification and development of improved waxy lines in terms of yield. Furthermore, the integration of genetic engineering approaches mediated editing of *waxy* gene and related regulatory elements, offers a powerful tool to fine-tune starch composition and functionality in a precise and predictable manner.

## Conclusions

This study identifies a novel waxy allele, *wx^e^*, with a corresponding molecular marker that allows direct comparison with the same WT background. The *wx^e^* allele also confers distinct grain quality traits, including reduced amylose content, enhanced kernel hardness, increased protein content, and an altered lipid profile providing a valuable genetic resource for future breeding. These improvements broaden the potential utility of waxy sorghum in specialty food products that demand unique starch characteristics, improved texture, and enhanced nutritional value. Also, the omics approaches helped in elucidating the complex regulatory landscape of starch biosynthesis in waxy sorghum. The observed changes in carbohydrate metabolism likely result from the loss of GBSS enzymatic activity, which disrupts the final step of amylose synthesis. The observed changes not only provide the genetic basis of endosperm development and carbohydrate metabolism but also offer promising targets for genetic manipulation. The knowledge generated from this study could be leveraged to enhance desirable seed composition traits, such as improved starch composition and processing qualities in sorghum.

## Data Availability

The RNA-seq data is available through National Center for Biotechnology Information-Sequence Read Archive (NCBI-SRA) under the Bio Project “PRJNA1222840.” The processed FPKM counts for all genes and differential expression analysis results (log₂ fold changes, *P*-values, q-values, and test statistic) for pairwise sample comparisons can be found in Supplementary Datasets 11 and 12, respectively submitted via the GSA figshare portal: https://doi.org/10.25387/g3.29630792. The data have been indexed in the NCBI Gene Expression Omnibus under accession number “GSE303394” to facilitate public access. Supplemental material available at *G3* online.
